# The taxonomic identity of three varieties of *Lecanorchis nigricans* (Vanilleae, Vanilloideae, Orchidaceae) in Japan

**DOI:** 10.3897/phytokeys.92.21657

**Published:** 2018-01-10

**Authors:** Kenji Suetsugu, Chie Shimaoka, Hirokazu Fukunaga, Shinichiro Sawa

**Affiliations:** 1 Department of Biology, Graduate School of Science, Kobe University, 1-1 Rokkodai, Nada-ku, Kobe, 657-8501, Japan; 2 Graduate School of Science and Technology, Kumamoto University, Kumamoto, Japan; 3 Tokushima-cho 3-35, Tokushima City, Tokushima, Japan

**Keywords:** Japan, *Lecanorchis nigricans* var. *yakusimensis*, *Lecanorchis nigricans* var. *patipetala*, *Lecanorchis taiwaniana*, lectotypification, mycoheterotrophy, taxonomy

## Abstract

To elucidate the taxonomy of the *Lecanorchis nigricans* Honda, 1931 species complex, the present study investigated the detailed morphology of three *L. nigricans* varieties in Japan. While L. nigricans var. patipetala Y.Sawa, 1980 and L. nigricans var. yakusimensis T.Hashim., 1990 have often been treated as synonyms of L. nigricans var. nigricans, the present study demonstrates that the three varieties are morphologically distinct. More specifically, L. nigricans var. nigricans only produces complete cleistogamous flowers and is distinct from the plants currently called “*L. nigricans*”, which are identical to the chasmogamous variety *L. nigricans* var. patipetala. The other chasmogamous variety L. nigricans var. yakusimensis can be easily distinguished from L. nigricans var. patipetala by its more spatulate tepals and higher cucullate lip. Therefore, the present study provides emended description of the three *L. nigricans* varieties based on type specimens and specimens collected from type localities. In addition, the isotype specimen of L. nigricans var. patipetala is designated as the lectotype because the holotype has been lost.

## Introduction

The genus *Lecanorchis* Blume, 1856 (Vanilleae, Vanilloideae, Orchidaceae) is a group of mycoheterotrophic plants that includes ca. thirty species and/or varieties (Hashimoto 1990; Szlachetko and Mytnik 2000; Govaerts et al. 2016; Suetsugu and Fukunaga 2016). Members of the genus are characterised by the presence of a calyculus, a cup-like structure located between the base of the perianth and the apex of the ovary (Hashimoto 1990) and are distributed across Southeast Asia, including India, Thailand, Laos, Vietnam, Malaysia, China, Taiwan, Japan, the Philippines, Indonesia, New Guinea and the Pacific Islands (Hashimoto 1990; Szlachetko and Mytnik 2000; Averyanov 2011).

Precise identification of *Lecanorchis* taxa is often hindered by the similar morphology and brief flowering periods (Hashimoto 1990; Averyanov 2005; Suddee and Pedersen 2011; Tsukaya and Okada 2013; Suetsugu and Fukunaga 2016; Suetsugu et al. 2016). In addition, important diagnostic characters are often lacking in herbarium specimens because the flowers of *Lecanorchis* members are easily dropped during preservation and the important diagnostic characters of some species have yet to be described in detail, especially for species that were first described many decades ago (reviewed by Suetsugu et al. 2016, 2017a, b). Therefore, adequate taxonomic studies of the genus have yet to be conducted (reviewed by Suetsugu et al. 2016, 2017a, b).

The taxonomic identity of *L. nigricans* Honda, 1931 has remained particularly unclear. The species was first described from Wakayama Prefecture (Kinki District, Japan; Honda 1931) and was subsequently reported from Taiwan, China, Thailand and Vietnam (Su 2000; Chen et al. 2009; Suddee et al. 2010; Hsu and Chung 2010; Vuong and Sridith 2016). Even though three *L. nigricans* varieties were described from Japan (Honda 1931; Sawa 1980; Hashimoto 1990), Yokota et al. (2016) defined the species in a broad sense, thereby synonymising L. nigricans var. patipetala Y. Sawa, 1980 and L. nigricans var. yakusimensis T. Hashim., 1990 as L. nigricans var. nigricans. However, it is possible that these treatments are based on the ambiguity of the original species description (Honda 1931; Sawa 1980; Hashimoto 1990) and that the species complex, in fact, comprises three entities.

To elucidate the taxonomy of the *L. nigricans* species complex, the present study investigated the detailed morphology of type specimens and specimens collected from type localities of three *L. nigricans* varieties in Japan. These findings revealed that the three varieties were morphologically distinct. More specifically, L. nigricans var. nigricans produces only complete cleistogamous flowers and is distinct from L. nigricans var. nigricans sensu Hashimoto 1990; Hashimoto et al. 1991; Nakajima and Ohba 2012; Yokota et al. 2016 (hereafter, the plants currently called “*L. nigricans*”), which is identical to the chasmogamous variety L. nigricans var. patipetala that was originally described from Kochi Prefecture (Shikoku District, Japan; Sawa 1980). In addition, the other chasmogamous variety L. nigricans var. yakusimensis, from Yakushima Island (Ryukyu Islands, Kagoshima Prefecture, Japan; Hashimoto 1990) can be distinguished from L. nigricans var. patipetala by its more spatulate tepals and higher cucullate lip. Therefore, the present study provides emended description of the three *L. nigricans* varieties based on type specimens and specimens collected from type localities.

## Materials and methods

### Morphological observation

In order to compare the morphologies of the three *Lecanorchis nigricans* varieties with previously recorded species, the authors reviewed the literature, conducted field sampling throughout Japan and examined both digitised plant specimens from online databases such as JSTOR Global Plants (http://plants.jstor.org/) and Plants of Taiwan (http://tai2.ntu.edu.tw/specimeninfo.php) and specimens from the following herbaria: TI, TNS, KYO, KPM, OSA, MBK, KOCH and KAG. Herbarium abbreviations follow Index Herbariorum (Thiers 2017, http://sweetgum.nybg.org/science/ih/). In total, at least 30 flowers were examined from 10 flowering plants to understand the morphological variations for each variety.

### DNA barcoding

For DNA isolation, the flowers of L. nigricans var. nigricans, L. nigricans var. patipetala, L. nigricans var. yakusimensis and their closely-related species *L. taiwaniana* S.S.Ying 1987 emend. Suetsugu, T.C. Hsu, S. Sawa, & Fukunaga 2016 were collected and desiccated in the field using silica gel (Table 1). DNA was extracted from these silica-dried plant materials, using the CTAB method (Wu et al. 2001). The rDNA internal transcribed spacer (ITS) region was amplified from the extracted DNA samples in 10 μL PCR mixtures that contained 2 μL extracted DNA, 0.05 µL TaKaRa Ex Taq Hot Start Version (Takara Bio, Japan), 10 μM of each primer (AB101 and AB102; Douzery et al. 1993), 0.25 μM of each dNTP and 1 μL 10× buffer, using an iCycler (BioRad, Japan) and the following conditions: initial denaturation at 94 °C for 5 min; followed by 30 cycles of 94 °C for 30 s, 55 °C for 30 s, and 72 °C for 1 min; followed by a final elongation at 72 °C for 7 min. The amplified PCR products were purified using EconoSpin (Gene Design, Inc.) columns and the subsequent samples were sent for sequencing to Eurofins Genomics (Ebersberg, Germany). The primers used for amplification were also used for sequencing.

**Table 1.** Lecanorchis spp. included in the current molecular analysis.

Taxon	Location	Collection date	Collection number	GenBank numbers
L. nigricans var. nigricans	Wakayama Pref., Kamitonda Town, Oka	20150819	H. Fukunaga s.n. OSA290835	LC315676
L. nigricans var. patipetala	Kochi Pref., Kochi City, Haruno	20150726	H. Fukunaga s.n. OSA290833	LC315674
L. nigricans var. yakusimensis	Kagoshima Pref., Yakushima Island, along Hanaage River	20150717	H. Yamashita s.n. OSA290834	LC315675
*L. taiwaniana*	Kochi Pref., Muroto City, Muroto Cape	20160816	H. Fukunaga s.n. OSA290836	LC315677

## Results and discussion

**Figure 1.**
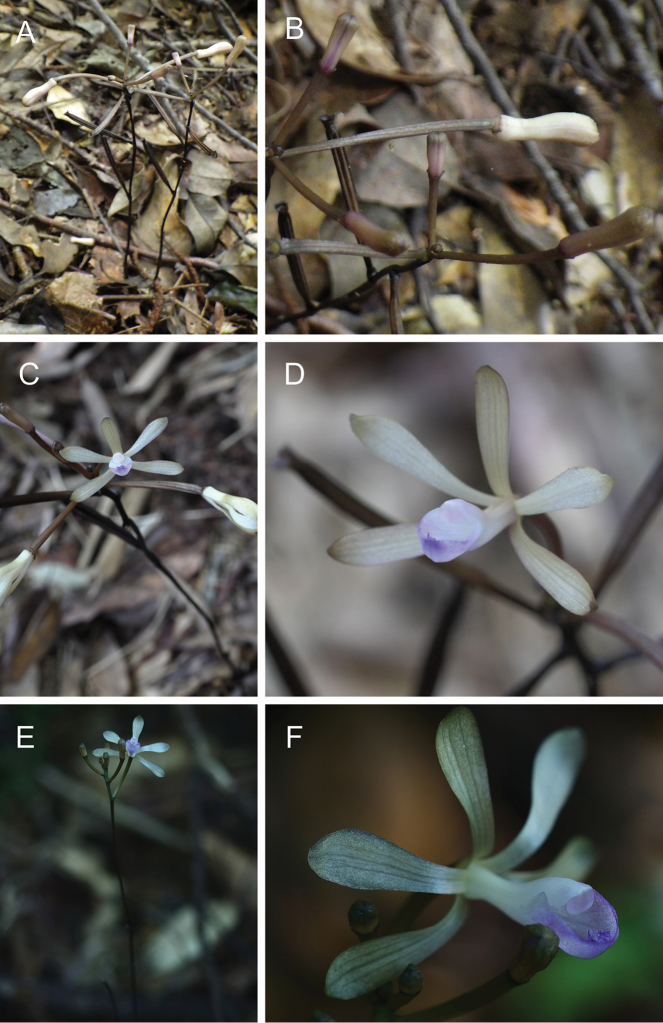
Photographs of three varieties of *Lecanorchis nigricans* in their natural habitats. **A** Flowering plant and **B** cleistogamous flowers of Lecanorchis nigricans var. nigricans in Oka, Kamitonda, Wakayama, Japan (its type locality). Photographed by Hirokazu Fukunaga **C** Flowering plant and **D** a flower of Lecanorchis nigricans var. patipetala in Haruno, Kochi, Japan. Photographed by Hisanori Takeuchi **E** Flowering plant and **F** a flower of Lecanorchis nigricans var. yakusimensis collected in Hanaage River, Yakushima, Japan (its type locality). Photographed by Hiroaki Yamashita.

**Figure 2.**
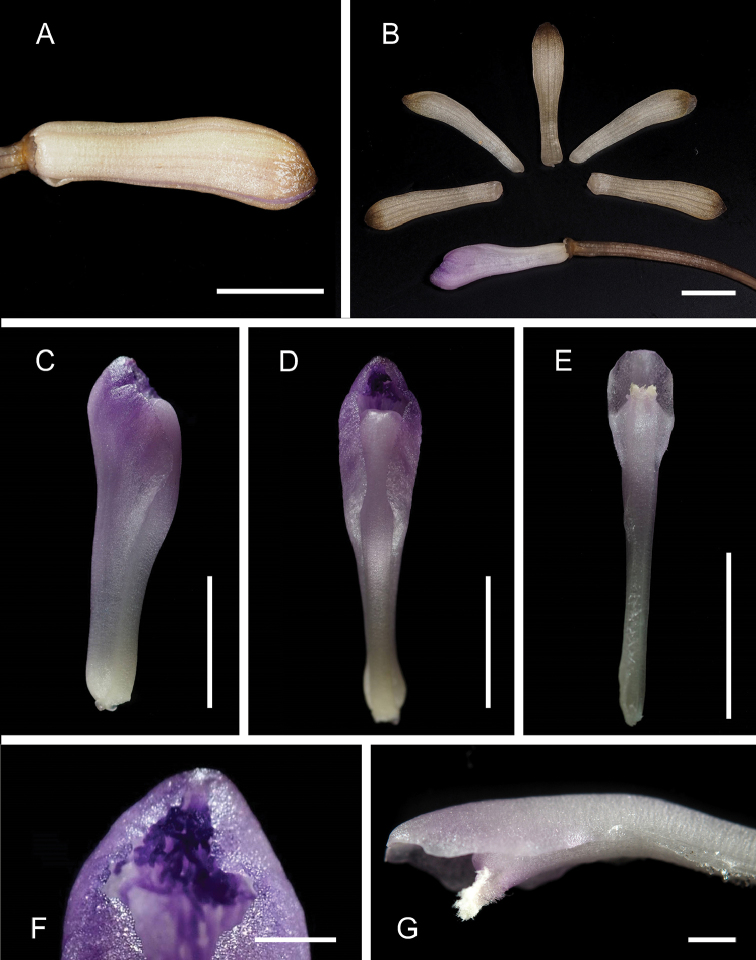
Dissected flowers of Lecanorchis nigricans var. nigricans collected in Konda, Kochi, Japan on 26 July 2015 (OSA). **A** Flower and pedicellate ovary **B** Dissected flower **C–D** Lip and column **E** Column **F** Hairs at anterior disc of lip **G** Glabrous status at the ventral side of the column. Scale bars: **A–E** 5 mm **F–G** 1 mm. Photographed by Takuto Shitara.

**Figure 3.**
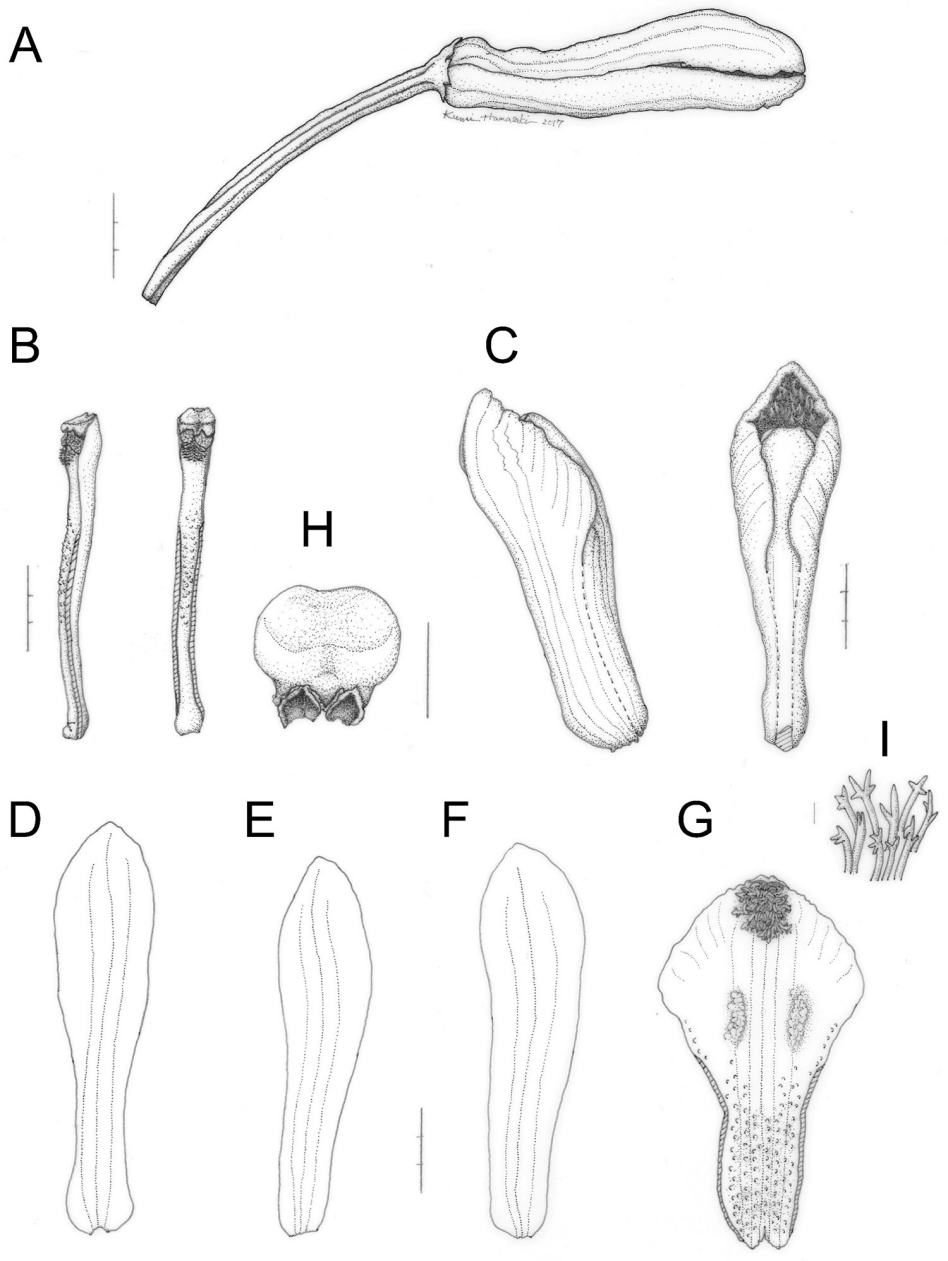
Lecanorchis nigricans var. nigricans collected in type locality on 3 August 2016 (OSA). **A** Flower and pedicellate ovary **B** Column **C** Lip and column **D** Dorsal sepal **E** Lateral petal **F** Lateral sepal **G** Flattened lip **H** Anther cap **I** Hairs at anterior disc of lip. Scale bars: **A–G** 3 mm **H** 1 mm **I** 0.1 mm. Line drawings by Kumi Hamasaki.

**Figure 4.**
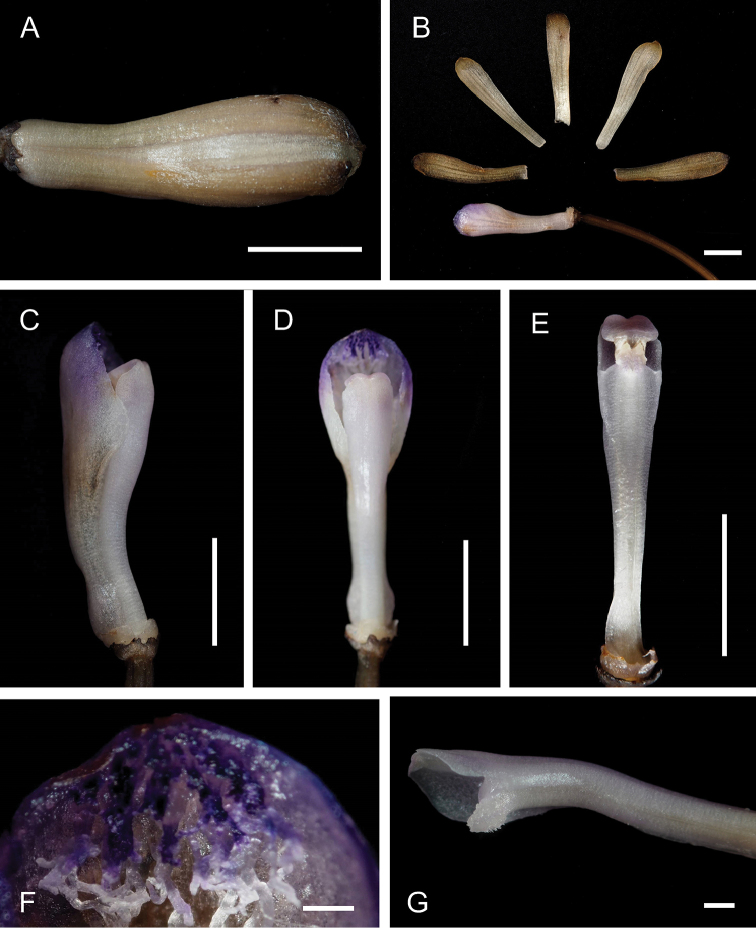
Dissected flowers of Lecanorchis nigricans var. patipetala collected in Haruno, Kochi, Japan on 26 July 2015 (OSA). **A** Flower and pedicellate ovary **B** Dissected flower **C–D** Lip and column **E** Column **F** Hairs at anterior disc of lip **G** Glabrous status at the ventral side of the column. Scale bars: **A–E** 5 mm **F–G** 0.5 mm. Photographed by Takuto Shitara.

**Figure 5.**
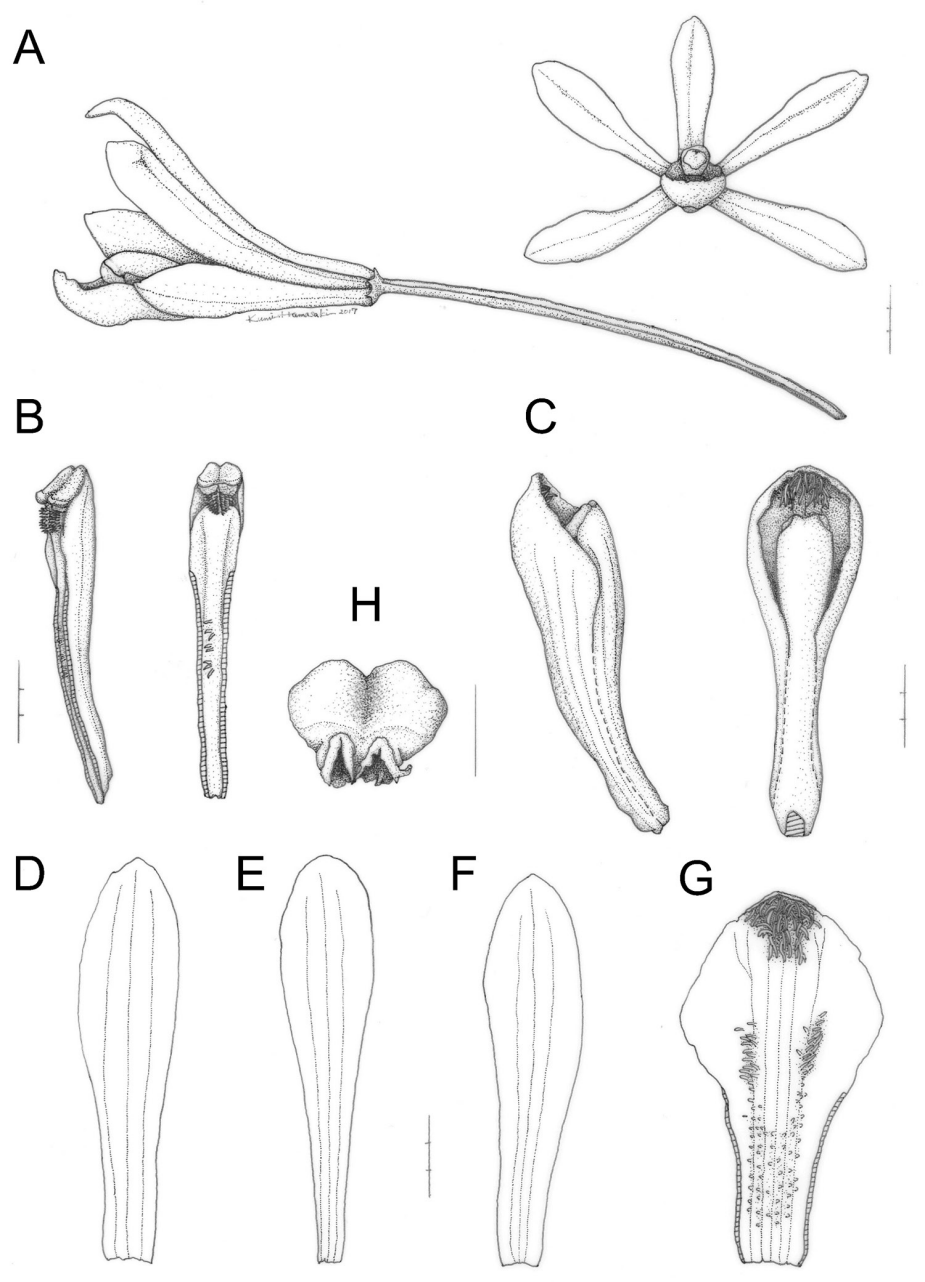
Lecanorchis nigricans var. patipetala collected in type locality on 28 July 2008 (OSA). **A** Flower and pedicellate ovary **B** Column **C** Lip and column **D** Dorsal sepal **E** Lateral petal **F** Lateral sepal **G** Flattened lip **H** Anther cap. Scale bars: **A–G** 3 mm **H** 1 mm. Line drawings by Kumi Hamasaki.

Even though Honda’s original description of *Lecanorchis nigricans* was insufficient in that most diagnostic characteristics were overlooked, he noted that neither the species’ sepals nor petals were open and that they, instead, were united, forming a cylindrical perianth tube (Honda 1931). This is quite different from the characteristics of the plants currently called “*L. nigricans*”, whose flowers are widely open (Hashimoto 1990; Hashimoto et al. 1991; Nakajima and Ohba 2012; Yokota et al. 2016). Indeed, the analysis of type specimens and specimens collected from type localities revealed that the flowers of L. nigricans var. nigricans remain completely closed throughout their flowering period (Table 2; Figs 1–3; https://www.youtube.com/watch?v=Y0SBE_J7bxo). The cleistogamous variety of *L. nigricans* was only distributed in limited areas of Wakayama (type locality), Miyazaki, Kochi, Tokushima and Tokyo (Hachijo Islands). Therefore, the same name should not be used for both the plants currently called “*L. nigricans*”, whose flowers are widely open and which is much more common throughout Japan (Hashimoto 1990; Hashimoto et al. 1991; Nakajima and Ohba 2012; Yokota et al. 2016). The chasmogamous variety of *L. nigricans* was initially described by Sawa (1980) as *L. nigricans* var. patipetala. It was found that there are no clear morphological differences amongst the plants currently called “*L. nigricans*”, L. nigricans var. patipetala lectotype specimens and L. nigricans var. patipetala type locality specimens (Table 2; Figs 4–5). Therefore, the name L. nigricans var. patipetala should be used for the common chasmogamous variety of *L. nigricans* that is found throughout Japan, with the exception of the Ryukyu Islands.

**Table 2.** Morphological characters of the three varieties of *Lecanorchis nigricans* and the morphologically similar *L. taiwaniana* and *L. tabugawaensis*.

Characters	L. nigricans var. nigricans	L. nigricans var. patipetala	L. nigricans var. yakusimensis	*L. tabugawaensis*	*L. taiwaniana*
Plant height	10–25(–30) cm	10–25(–30) cm	10–25(–30) cm	15–45 cm	15–45 cm
Rachis color in developing stage	purplish white	purplish white	purplish white	yellowish white	yellowish white
Rachis color in fruiting stage	black	black	black	brownish black	brownish black
Rachis length	2–8 cm	2–8 cm	2–8 cm	6–15 cm	(2–)6–15 cm
Internode length of upper half of rachis	1–3 mm	1–6(–10) mm	1–6(–10) mm	5–15 mm	5–15 mm
Floral condition	cleistogamous	chasmogamous	chasmogamous	chasmogamous	chasmogamous
Sepal and petal color	purplish white	purplish white	purplish white	yellowish white tinged with light purple	yellowish white tinged with light purple
Width of sepal and lateral petal	2.8–3.7 mm	3.0–3.8 mm	3.3–4.0 mm	2.0–2.5mm	2.0–2.5(–3.0) mm
Shape of sepal and lateral petal	oblong-oblanceolate	oblong-oblanceolate	oblanceolate-spatulate	oblong	oblong
Lip shape	indistinctly 3-lobed	almost entire	almost entire and cucullate	almost entire	indistinctly 3-lobed
Colored area in lip	ca. apical 1/3–1/2	ca. apical 1/3–1/5	ca. apical 1/3	ca. apical more than 2/3	ca. apical 1/4–1/5
Proportion of the column fusion with lip	ca. 1/2	ca. 1/2	ca. 1/2	2/5–1/2	3/5–2/3
Column shape	slightly recurved	recurved	strongly recurved	straight	recurved
Apical part of the adaxial lip surface	dense, short and frequently branched hairs	scarce, long and rarely branched hairs	scarce, long and rarely branched hairs	glabrous	scarce, long and rarely branched hairs
Pubescence at basal part of column	glabrous	glabrous to slightly hairy	densely hairy	glabrous	densely hairy
Capsule color	black	black	black	bright brown	bright brown
Angle between capsule and inflorescence axis	70–90°	70–90°	70–90°	20–45°	20–45°

Data of the related species from Suetsugu et al. (2016) and Suetsugu and Fukunaga (2016)

Detailed morphological investigation revealed that L. nigricans var. patipetala could also be distinguished from L. nigricans var. nigricans by its larger perianth tube (14–17 mm vs. 11–14 mm), the shorter coloured area of its lip (ca. apical 1/3–1/5 vs. ca. apical 1/2–1/3), the shape of its lip apex in the natural situation (broadly rounded vs. acute), the status of lip hairs near apex (scarce, long and rarely branched multicellular hairs vs. dense, short and frequently branched multicellular hairs), the shape of the column (recurved vs. slightly recurved), the width of its petal base (narrow ca. 1.0–1.3 mm vs. relatively wide ca. 1.5–2.5 mm) and the shape of its anther cap (strongly bilobed v.s. slightly bilobed; Table 2; Figs 1–5).

**Figure 6.**
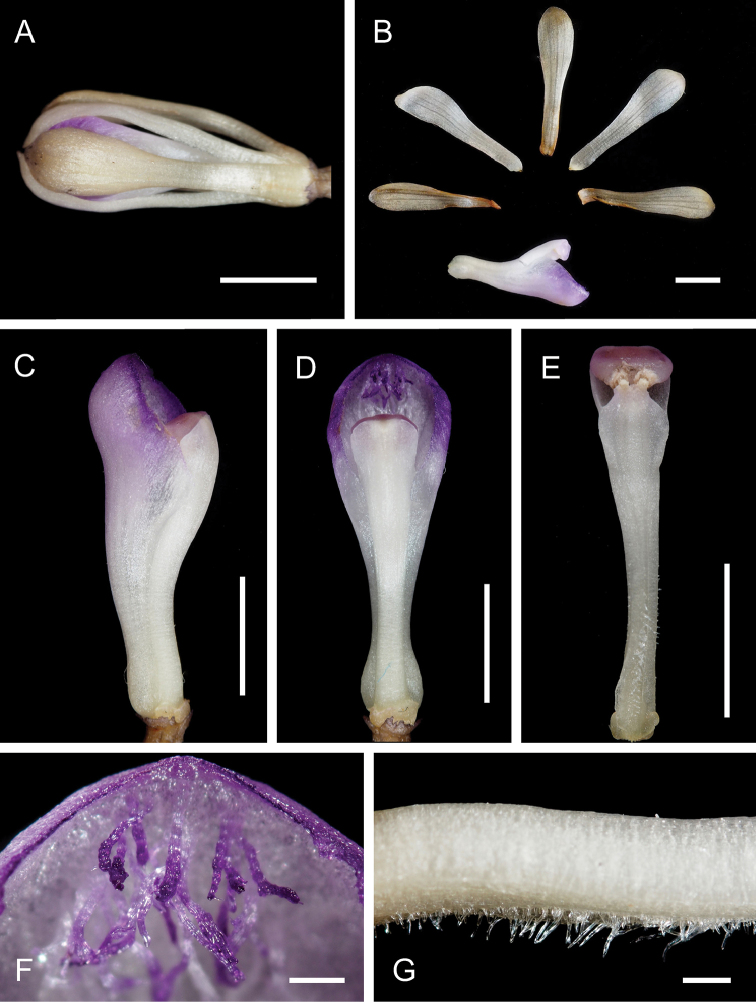
Dissected flowers of Lecanorchis nigricans var. yakusimensis collected in type locality on 17 July 2015 (OSA). **A** Flower and pedicellate ovary **B** Dissected flower **C–D** Lip and column **E** Column **F** Hairs at anterior disc of lip **G** Pubescence at the ventral side of the column. Scale bars: **A–E** 5 mm **F–G** 0.5 mm. Photographed by Takuto Shitara.

**Figure 7.**
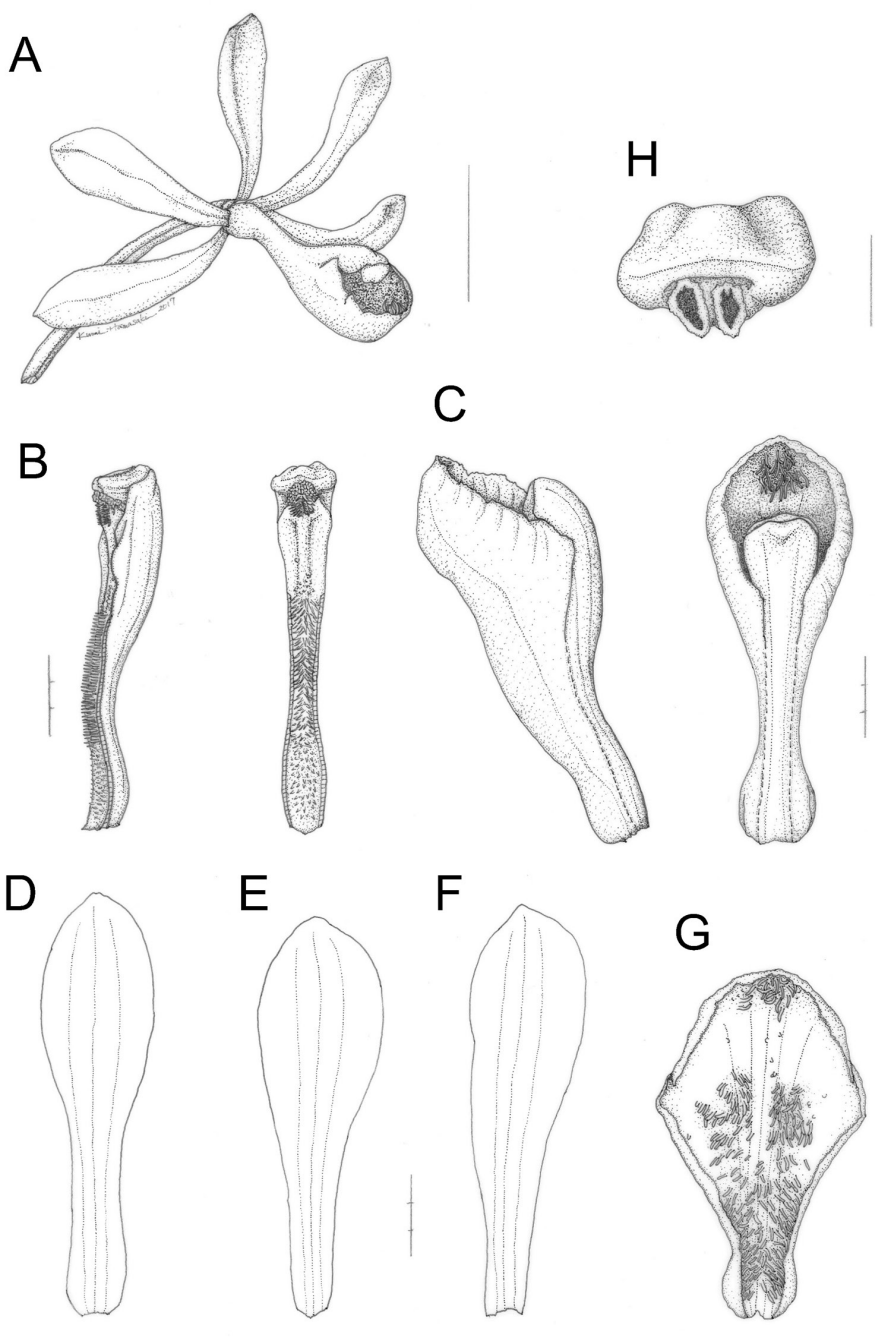
Lecanorchis nigricans var. yakusimensis collected in type locality on 17 July 2015 (OSA). **A** Flower and pedicellate ovary **B** Column **C** Lip and column **D** Dorsal sepal **E** Lateral petal **F** Lateral sepal **G** Flattened lip **H** Anther cap. Scale bars: **A** 1 cm **B–G** 3 mm **H** 1 mm. Line drawings by Kumi Hamasaki.

In addition, the other chasmogamous variety L. nigricans var. yakusimensis was described from Yakushima Island (Ryukyu Islands, Japan). However, even though pubescence at the ventral side of the column was highlighted as the variety’s diagnostic character (Hashimoto 1990; Hashimoto et al. 1991), the column of L. nigricans var. patipetala also varies from glabrous to slightly hairy. Nonetheless, the column of L. nigricans var. patipetala is less hairy than that of *L. nigricans* var. yakusimensis. In addition, L. nigricans var. yakusimensis possesses more spatulate sepals and petals, as well as more highly cucullate lips, whereas L. nigricans var. patipetala possesses more oblong sepals and less cucullate lips. Furthermore, L. nigricans var. yakusimensis can be distinguished from L. nigricans var. patipetala by its wider anther caps (ca. 2.0 mm. vs. ca. 1.5 mm) and more recurved column. Thus, L. nigricans var. yakusimensis can be distinguished not only by its more hairy column, but also its tepals, lip, anther cap and column shape (Table 2; Figs 4–7).

As L. nigricans var. yakusimensis is more common than L. nigricans var. patipetala in both Yakushima and Taiwan (Suetsugu and Hsu, unpublished data), it is likely that the variety is also distributed on the other Ryukyu Islands. Thus, *L. oligotricha* Fukuy. 1942 that has been described from Iriomote Island (Ryukyu Islands), may actually be identical to L. nigricans var. yakusimensis, while *L. oligotricha* has been considered as a synonym of L. nigricans var. nigricans. Even so, the name *L. nigricans* var. *yakusimensis* is preferred because the taxon should be recognised as an intraspecific variety, instead of an independent species. In addition, it should be noted that some *L. nigricans* specimens from the Ryukyu Islands were misidentifications of *L. taiwaniana*. However, it is unlikely that *L. oligotricha* is synonymous with *L. taiwaniana*, owing to differences in sepal and petal shape, according to the protologue (Fukuyama 1942). Unfortunately, the type materials of *L. oligotricha* in KPM are poorly preserved and no mature flowers are available for dissection (Inoue et al. 1998). Therefore, further investigation of *L. oligotricha* specimens from the species’ type locality will be critical to clarifying the species’ taxonomic status.

Based on the findings of the present study, it is suggested that the two varieties L. nigricans var. yakusimensis and L. nigricans var. patipetala should be revived since the distinct morphological characteristics of the three varieties are clear and stable. It is also considered that the aforementioned differences amongst the three varieties are relatively minor and represent interspecific variation. The identical DNA barcode sequences of *L. nigricans*, L. nigricans var. patipetala and L. nigricans var. yakusimensis also support this conclusion, whereas the sequence divergence of the three *L. nigricans* varieties and *L. taiwaniana* (i.e. 5 substitutions) support the independent specific status of both *L. nigricans* and *L. taiwaniana*, even though the two are sometimes considered synonymous (e.g. Su 2000). Actually, *L. taiwaniana* and its closely-related species *L. tabugawaensis* Suetsugu & Fukunaga 2016 can easily be distinguished from the three varieties of *L. nigricans* by having taller inflorescences, longer and lighter coloured rachis, yellowish-white, narrower sepals and petals and brighter brown suberect capsules (Suetsugu and Fukunaga 2016; Suetsugu et al. 2016).

In addition, *L. nigricans* has recently been reported from China, Thailand and Vietnam (Su 2000; Chen et al. 2009; Suddee et al. 2010; Vuong and Sridith 2016). Vuong and Sridith (2016) noted that the specimens collected from Vietnam possessed character traits that were intermediates of those of *L. nigricans* and *L. taiwaniana*. The various morphological character of *L. nigricans* other than from Japan would actually imply that more than one taxon was placed under the name *L. nigricans* and that further clarification may be needed in these regions.

## Taxonomic treatment

### Lecanorchis nigricans

Taxon classificationPlantaeORDOFAMILIA

Honda emend. Suetsugu & Fukunaga

Figs 2 , 3

#### Type.

JAPAN, Wakayama Pref., Nishimuro County, Iwata, Oka, date unknown 1931, *K. Kashiyama s.n.* (holotype TI!; Isotype TI!).

#### Emended description.

Terrestrial, mycoheterotrophic herb. Inflorescence 10–25(–30) cm tall, solitary or branched at lower-half, white at flowering, black at fruiting, glabrous, ca. 0.8–2.0 mm in diam., with scale-like sheaths. Rhizome erect, J-shaped or complex, ligneous. Roots simple, radiate numerous, horizontally or downward elongate to 20–30 cm long, yellowish brown. Rachis 2–8 cm, 3–15 flowered, internode length of upper-half of rachis, 1–3 mm. Floral bracts triangular, acute, 1.0–2.0 mm long. Pedicellate ovary ascending, 15–25 mm long. Flowers enclosed or never opening. Sepals purplish white, linear, oblong-oblanceolate, ca. 11–14 mm long, 3.0–3.7 mm wide, apex obtuse. Petals purplish white, linear, oblong-oblanceolate, 13–14 mm long, 2.8–3.6 mm wide, apex obtuse. Lip spatulate, strongly 12–14 mm long, 3.2–3.9 mm wide in a natural situation, ca. 6.5–7.5 mm wide when flattened, disc with rather dense, short multicellular hairs which are often branched, near apex, or acute at apex in a natural situation. Column 10–12 mm long, 1.1–2.0 mm wide slightly recurved, fused with lip about 1/2 its length, ventrally glabrous or slightly puberulent; anther purplish white, ca. 1.5 mm wide. Capsule 17–30 mm long, cylindrical-fusiform, black, ascending at 70–90 degree angle from the axis. Flowering in late-June to mid-September.

#### Additional specimens examined.

JAPAN. Miyazaki Pref.: Nishimorokata County, Takaharu Town, 29 July 2016, *Nobuyuki Inoue s.n.* (OSA), JAPAN. Wakayama Pref.: Kamitonda Town, 3 Aug. 2016, *H. Fukunaga s.n.* (OSA, in spirit collection), Kamitonda Town, Oka 19 Aug. 2015, *H. Fukunaga s.n.* (MBK, in spirit collection), Nishimuro County, Iwata, Oka, 27 May 1929, *K. Kashiyama s.n.* (KYO), Nishimuro County, Iwata, Oka, 27 July 1929, *K. Kashiyama s.n.* (TI, KYO), Iwata, Oka, 13 July 1930, *K. Kashiyama s.n.* (KYO), Nishimuro County, Iwata, Oka, 14 July 1931, *K. Kashiyama s.n.* (TI), Nishimuro County, Iwata, Oka, 4 Aug. 1931, *K. Kashiyama s.n.* (TI), Nishimuro County, Iwata Village, August 1931, collector unknown (TSN), Nishimuro County, Iwata, Oka, 20 Sept. 1932, *K. Kashiyama s.n.* (KYO), Nishimuro County, Iwata, Oka, 20 Sept. 1933, *K. Kashiyama s.n.* (TI), Nishimuro County, Iwata, Oka, 1 Aug. 1933, *S. Kitasima s.n.* (KYO), Kamitonda Town, Oka 28 July 1984. *S. Kashiyama 911* (MBK, in spirit collection), JAPAN. Kochi Pref.: Kochi City, Zigokudani 29 July 1978. *Y. Sawa s.n.* (MBK, in spirit collection), Kochi Pref., Kochi City, Zigokudani, 8 Aug. 1978, *Yutaka Sawa O-86* (TI), Kochi City, Kouda, 26 July 2015, *Hirokazu Fukunaga s.n.* (OSA, in spirit collection), Kami County, Tosayamada Town, Aburaishi, 12 Aug. 1986, *Yutaka Sawa 1138* (TI), Kami County, Tosayamada Town, Aburaishi, 12 Aug. 1986, *Yutaka Sawa 1142* (TI), Kami City, Kahoku Town, 25 July 2016, *Hisanori Takeuchi s.n.* (OSA), JAPAN. Tokushima Pref.: Kaifu-County, Kainan Town, 18 July 1977. *T. Nakagawa 1043* (MBK), JAPAN. Tokyo Metropolis: Izu Islands, Hachijo Island. 6 Dec. 1940, *J. Ohchi s.n.* (TI), Izu Islands, Hachijo Island. 28 July 2016, *Masayuki Ishibashi s.n.* (OSA).

### Lecanorchis nigricans Honda var. patipetala

Taxon classificationPlantaeORDOFAMILIA

Y.Sawa emend. Suetsugu & Fukunaga

Figs 4 , 5

#### Type.

JAPAN, Kochi Pref., Kochi City, Ikku, 5 Aug.1979, *Y. Sawa O-101* (lectotype designated here, MBK-0022411)

#### Synonym.

*Lecanorchis nigricans auct. non* Honda (1931: 470): Hashimoto (1990:27), Hashimoto et al. (1991: 119), Nakajima and Ohba (2012: 141), Yokota et al. (2016: 248), syn. nov.

#### Emended description.

Terrestrial, mycoheterotrophic herb. Inflorescence 10–25(–30) cm tall, solitary or branched at lower-half, purplish-white at flowering, black at fruiting, glabrous, ca. 0.8–1.5 mm in diam., with scale-like sheaths. Rhizome erect, J-shaped or complex, ligneous. Roots simple, radiate numerous, horizontally or downward elongate to 20–30 cm long, yellowish brown. Rachis 2–8 cm, 3–15 flowered, internode length of upper-half of rachis, 1–6(–10) mm. Floral bracts triangular, acute, 0.8–3.0 mm long. Pedicellate ovary ascending, 15–30 mm long. Flowers widely opening, ca. 2.5 cm in diameter. Sepals purplish white, linear, oblong-oblanceolate, ca. 12–17 mm long, 2.7–3.4 mm wide, apex obtuse. Petals purplish white, linear, oblong-oblanceolate, 13–17 mm long, 2.6–3.4 mm wide, apex obtuse. Lip shallowly spatulate, strongly 13–15 mm long, 4.0–4.5 mm wide in natural situation, ca. 6.0–7.0 mm wide when flattened, disc with rather scarce, long multicellular hairs which are rarely branched, near apex. Column 10–13 mm long, 1.2–2.8 mm wide slightly recurved, fused with lip about 1/2 its length, ventrally glabrous or slightly puberulent; anther white, ca. 1.5 mm wide. Capsule 15–30 mm long, black, cylindrical-fusiform, ascending at 70–90 degree angle from the axis. Flowering in mid-July to mid-September.

#### Note.

When describing L. nigricans var. patipetala, Sawa (1980) cited the specimens that he had collected from Ikku (Kochi Prefecture) on 5 August 1978. However, even though Sawa reported that the holotype specimen had been deposited in MBK and that the isotype specimens had been deposited in KYO and KOCH, no specimens fitting Sawa’s description could be located, despite intensive surveys of MBK, KYO and KOCH. The only putative original specimen that was found was a specimen in MBK that was collected by Sawa from Ikku (Kochi Prefecture) on 5 August 1979. This specimen has already been treated as an isotype by the MBK curator. The status of the specimen is somewhat controversial since both the collection date (5 August 1979 vs. 5 August 1978) and collection number (*O-101* vs. *O-135*) differ from those of the L. nigricans var. patipetala protologue. However, the MBK specimen should still be recognised as an L. nigricans var. patipetala isotype, because the collection dates are similar enough that the difference could be regarded as a typing error. Actually, Hashimoto (1990), who investigated the L. nigricans var. patipetala holotype when the specimen was still preserved in MBK, cited the collection date as 5 August 1979 and collection number *O-101*. Actually, the collection number *O-101* was cited as a collection number for the *Gastrodia pubilabiata* holotype in the paper by Sawa (1980) that described both L. nigricans var. patipetala and *G. pubilabiata*. It is highly possible that the collection number for L. nigricans var. patipetala and *G. pubilabiata* was somehow reversed in the protologue. Therefore, in order to stabilise the taxonomic status of L. nigricans var. patipetala, the MBK isotype was designated as the lectotype, according to Articles 9.11 and 9.12 of the ICN (McNeill et al. 2012).

#### Additional specimens examined.

JAPAN. Nagasaki Pref.: Fukuejima Island, Goto City, Kishiku Town, 24 July 2017, *Ueda Kouichi s.n.* (OSA, in spirit collection), Fukuejima Island, Goto City, Tomie Town, 21 July 2017 *Ueda Kouichi s.n.* (OSA, in spirit collection), JAPAN. Kochi Pref.: Kochi City, Engyouji, 4 Aug. 1979, *Yutaka Sawa O-95* (MBK), Kochi City, Ikku, 31 July 1978, *Yutaka Sawa O-83* (MBK), Kochi City, Ikku, 13 Aug. 1980, *Yutaka Sawa O-105* (MBK), Kochi City, Ikku, 5 Aug. 1980, *Yutaka Sawa O-103* (MBK), Kochi City, Haruno, 26 July 2015, *Hirokazu Fukunaga s.n.* (OSA, in spirit collection), Kochi City, Engyozi, 4 Aug. 1979, *Yutaka Sawa O-91* (TI), Kochi City, Engyozi, 31 July 1979, *Yutaka Sawa O-89* (TI), Kochi City, Engyozi, 31 July 1979, *Yutaka Sawa O-90* (TI), Kochi City, Ikku, 13 July 1980, *Shinichiro Sawa O-106* (TI), Kochi City, Ikku, 2 July 1983, *Yutaka Sawa 671* (TI), Kochi City, Ikku, 2 July 1983, *Yutaka Sawa 944* (TI), Kochi City, Ikku, 28 July 2007, *Hirokazu Fukunaga s.n.* (OSA, in spirit collection), Kochi City, Engyouji, 31 July 1979, *Yutaka Sawa s.n.* (MBK, in spirit collection), Kochi City, Engyouji, 4 Aug. 1979, *Yutaka Sawa s.n.* (MBK), Kochi City, Ikku, 5 Aug. 1978, *Yutaka Sawa O-102* (MBK), JAPAN. Mie Pref.: Ise City, Mt. Kamiji, 30 Oct. 1982, *Hiroshi Hara s.n.* (TI), Ise City, Mt. Kamiji, 23 July, *Chizuru Chuma s.n.* (TI), JAPAN. Aichi Pref.: Toyohashi City, Ooiwa Town, 11 Aug. 2000, *Yutaka Yoshida s.n.* (MBK, in spirit collection), JAPAN. Shizuoka Pref.: Inasa County, Hosoe Town, 31 Aug. 1981. *Yutaka Sawa* O-387 (MBK, in spirit collection), Inasa County, Hosoe Town, 31 Aug. 1981. *Yutaka Sawa* O-388 (MBK), Inasa County, Hosoe Town, along Hamanako, 9 Aug. 1981. *Isamu Yamashita 387* (MBK), Osuka Town, Kawaramachi, 15 Aug. 1982. *Takao Sugino 812* (MBK), Toyooka Village, Dairakuji, 13 Aug. 1982. *Takao Sugino 819* (MBK), Mori Town, Daimon, 13 Aug. 1982. *Takao Sugino 818* (MBK), Shimoda City, Mt. Nesugata, ca. 170 m alt, 12 Aug. 1989, *J. Kanda & Y. Hanei s.n.* (TNS), Iwata County, Toyooka Village, Dairakuji, 30 July 1982, *Yoshifusa Kurosawa 58* (TSN), Iwata County, Toyooka Village, Dairakuji, 8 Aug. 1978, *Yoshifusa Kurosawa s.n.* (TSN), Hamamatsu City, Takizawa Town, Higashiyama, 9 Aug. 1981, *Isamu Yamashita 377* (MBK), Atami City, Momoyama Town, 1 Sept. 1975, *Sunao Kikuchi s.n.* (KYO), Iwata City, Shikiji, 22 1979, *T. Tuyama s.n.* (TI, KYO), JAPAN. Kanagawa Pref.: Yokosuka City, *Saburo Takahashi s.n.*, 27 July 1989 (KPM), Tsukui County, Shiroyama Town, Ohdo, *F. Yamazaki s.n.*, 30 Aug. 1992 (TI), JAPAN. Tokyo Metropolis: Hachioji City, *Tsuneo Asama s.n.*, date unknown, July 1981 (KPM), Hachioji City, *Hirokazu Fukunaga & Gen Gomi s.n.*, 23 Aug. 2008 (MBK, in spirit collection), JAPAN. Chiba Pref.: Kimitsu City, Toyofusa Island, *Shigeki Fukushima s.n.*, 13 Aug. 2016 (OSA, in spirit collection), JAPAN. Chiba Pref., Abo County, Maruyama Town, 17 August 1989, *Joju Haginiwa JH015366* (TSN), JAPAN. Chiba Pref., Abo County, Maruyama Town, 17 Aug. 1989, *Joju Haginiwa JH015367* (TSN), JAPAN. Chiba Pref., Abo County, Maruyama Town, 17 Aug. 1989, *Joju Haginiwa JH015368* (TSN), Abo County, Maruyama Town, 17 Aug. 1989, *Joju Haginiwa JH015369* (TSN), Abo County, Maruyama Town, 17 Aug. 1989, *Joju Haginiwa JH015370* (TSN), Abo County, Maruyama Town, 17 Aug. 1989, *Joju Haginiwa JH015371* (TSN), Abo County, Maruyama Town, 17 Aug. 1989, *Joju Haginiwa JH015372* (TSN), Abo County, Maruyama Town, 17 Aug. 1989, *Joju Haginiwa JH038908* (TSN), Abo County, Maruyama Town, 17 Aug. 1989, *Joju Haginiwa JH038909* (TSN), Abo County, Maruyama Town, 17 Aug. 1989, *Joju Haginiwa JH040541* (TSN), JAPAN. Ibaraki Pref.: Hitachi City, Okubo Town, near Umegaoka Hospital, ca. 170 m alt, 25 Aug, 25. 1990, *T. Hashimoto s.n.* (TNS), Tsukuba City, Mt. Tsukuba, 7 Aug. 2007, *S. Matsumoto 070807-9* (TSN).

### Lecanorchis nigricans Honda var. yakusimensis

Taxon classificationPlantaeORDOFAMILIA

T.Hashim. emend. Suetsugu & Fukunaga

Figs 6 , 7

#### Type.

Japan, Kagoshima Pref., Yakushima Island, along the Hanaage River, 21–27 July 1986, *Y. Hanei s.n.* (holotype TNS!).

#### Emended description.

Terrestrial, mycoheterotrophic herb. Inflorescence 10–25(–30) cm tall, solitary or branched at lower-half, purplish-white at flowering, black at fruiting, glabrous, ca. 0.8–1.5 mm in diam., with scale-like sheaths. Rhizome erect, J-shaped or complex, ligneous. Roots simple, radiate numerous, horizontally or downward elongate to 20–30 cm long, yellowish brown. Rachis 2–8 cm, 3–15 flowered, internode length of upper-half of rachis, 1–6(–10) mm. Floral bracts triangular, acute, 0.7–2.0 mm long. Pedicellate ovary ascending, 14–30 mm long. Flowers widely opening, ca. 2.5 cm in diameter. Sepals purplish white, linear, oblanceolate-spatulate, ca. 13–17 mm long, 3.3–4.0 mm wide, apex obtuse. Petals purplish white, linear, oblanceolate-spatulate, 13–17 mm long, 3.3–4.0 mm wide, apex obtuse. Lip spatulate to cucullate, strongly concave, 12–15 mm long, ca. 4.5 mm wide in natural situation, 7.5–8.0 mm wide when flattened, disc with rather scarce, long multicellular hairs which are rarely branched, near apex. Column 10–13 mm long, recurved, fused with lip about 1/2 its length, ventrally densely puberulent; anther purplish white, ca. 2.0 mm wide. Capsule 20–30 mm long, cylindrical-fusiform, black, ascending at 70–90 degree angle from axis. Flowering in mid-July to mid-September.

#### Additional specimens examined.

JAPAN. Kagoshima Pref., Yakushima Island: along Hanaage River, 17 July 2015, *Hiroaki Yamashita s.n.* (OSA, in spirit collection), along Hanaage River, 27 July 2017, *Hiroaki Yamashita s.n.* (OSA, in spirit collection), along Osaki River, 17 July 2015, *Hiroaki Yamashita s.n.* (OSA, in spirit collection), along Nakase River, 17 July 2015, *Hiroaki Yamashita s.n.* (OSA, in spirit collection), Haruhira, 17 July 2015, *Hiroaki Yamashita s.n.* (OSA, in spirit collection), along Futamata River, Mt. Mochomu, 26 July 1991, *Yoshie Hanei s.n.* (TSN), along Futamata River, Mt. Mochomu, 26 July 1991, *Yoshie Hanei s.n.* (TSN), Kurio, 5 Aug. 1975, *Doi s.n.* (TSN).

## Supplementary Material

XML Treatment for Lecanorchis nigricans

XML Treatment for Lecanorchis nigricans Honda var. patipetala

XML Treatment for Lecanorchis nigricans Honda var. yakusimensis
